# Perceived Statistical Knowledge Level and Self-Reported Statistical Practice Among Academic Psychologists

**DOI:** 10.3389/fpsyg.2018.00996

**Published:** 2018-06-22

**Authors:** Laura Badenes-Ribera, Dolores Frias-Navarro, Nathalie O. Iotti, Amparo Bonilla-Campos, Claudio Longobardi

**Affiliations:** ^1^Departament de Metodologia de les Ciències del Comportament, Universitat de València, Valencia, Spain; ^2^Dipartimento di Psicologia, Università degli Studi di Torino, Turin, Italy

**Keywords:** effect size, confidence interval, meta-analysis, survey study, education

## Abstract

**Introduction:** Publications arguing against the null hypothesis significance testing (NHST) procedure and in favor of good statistical practices have increased. The most frequently mentioned alternatives to NHST are effect size statistics (ES), confidence intervals (CIs), and meta-analyses. A recent survey conducted in Spain found that academic psychologists have poor knowledge about effect size statistics, confidence intervals, and graphic displays for meta-analyses, which might lead to a misinterpretation of the results. In addition, it also found that, although the use of ES is becoming generalized, the same thing is not true for CIs. Finally, academics with greater knowledge about ES statistics presented a profile closer to good statistical practice and research design. Our main purpose was to analyze the extension of these results to a different geographical area through a replication study.

**Methods:** For this purpose, we elaborated an on-line survey that included the same items as the original research, and we asked academic psychologists to indicate their level of knowledge about ES, their CIs, and meta-analyses, and how they use them. The sample consisted of 159 Italian academic psychologists (54.09% women, mean age of 47.65 years). The mean number of years in the position of professor was 12.90 (*SD* = 10.21).

**Results:** As in the original research, the results showed that, although the use of effect size estimates is becoming generalized, an under-reporting of CIs for ES persists. The most frequent ES statistics mentioned were Cohen's *d* and R^2^/η^2^, which can have outliers or show non-normality or violate statistical assumptions. In addition, academics showed poor knowledge about meta-analytic displays (e.g., forest plot and funnel plot) and quality checklists for studies. Finally, academics with higher-level knowledge about ES statistics seem to have a profile closer to good statistical practices.

**Conclusions:** Changing statistical practice is not easy.This change requires statistical training programs for academics, both graduate and undergraduate.

## Introduction

In the past 3 years, there has been increasing criticism of the null hypothesis significance testing procedure (NHST) based on *p*-values and the dichotomous decision to maintain or reject the null hypothesis (e.g., Nuzzo, 2014; Trafimow and Marks, 2015; Allison et al., 2016).

Most of the criticism refers to misconceptions about what the results stemming from an NHST mean (e.g., the false belief that the *p*-value is an indicator of the practical importance of the findings), and the NHST “ritual” ends with the binary decision to reject/not reject the null hypothesis (Badenes-Ribera et al., 2015; Perezgonzalez, 2015). In addition, the quest for the magical “*p* < 0.05” leads to bad statistical practices (e.g., *p*-hacking) that distort scientific knowledge and harm scientific progress (Fanelli, 2009; Gadbury and Allison, 2014; Wicherts et al., 2016; Agnoli et al., 2017).

Given these misconceptions about the *p*-value and the misuse of the NHST procedure, the American Psychological Association (American Psychological Association 2010; Appelbaum et al., 2018), the American Educational Research Association (2006), the American Statistician Association (ASA) (Wasserstein and Lazar, 2016), the Open Science Collaboration (2015), the *new statistic* approach (e.g., Cumming, 2012; Kline, 2013), and many editors of psychology journals (e.g., Eich, 2014) make several recommendations. For example, they recommend reporting effect size statistics (ES) and their confidence intervals (CIs), in an attempt to reduce overreliance on NHST and dichotomous decisions (e.g., Maxwell et al., 2015; Krueger and Heck, 2017).

An effect size represents the strength or magnitude of a relationship between the variables in the population, or a sample-based estimate of that quantity (Cohen, 1988). Effect sizes (ESs) provide more information than the *p*-value because they address the question, “How large is the relationship or difference?” Therefore, they can help to interpret whether the effect observed in a study is large enough to be theoretically or practically important. ES can be reported in raw units (e.g., means, differences between two means, proportions) or in some standardized units (Cumming, 2014). Raw units provide a useful estimate of effect size when the measures are reported on a meaningful scale or well-known due to widespread use (e.g., IQ), and they can be used to compare studies that employ the same scale (Fritz et al., 2012). Standardized ES can be useful for comparing studies and conducting meta-analyses. Thus, meta-analyses frequently focus on the simple correlation between the independent and dependent variables as standardized ES input (Borenstein et al., 2009). Dozens of standardized ES can be found. Overall, two main groups can be distinguished: ES that use the standardized group mean difference, such as Cohen's *d*, Glass's *g*, Hedges's *g*_u_, and Cohen's *f*; and ES based on the percentage of explained variance or the correlations between variables, such as *r, R*^2^*/r*^2^, η^2^*, w*^2^ (Rosnow and Rosenthal, 2009).

The most frequently reported ES measures in psychological journals are *r, R*^2^, Cohen's *d*, and η^2^ (Sun et al., 2010; Lakens, 2013; Peng et al., 2013). However, these measures have been criticized for being biased (e.g., they tend to be positively biased), for lack of robustness in the case of outliers, and for instability when statistical assumptions are violated (Wang and Thompson, 2007; Grissom and Kim, 2012; Kline, 2013).

The CI for a parameter (e.g., effect size) defines a range of values generated by a procedure that, in repeated-sampling, has a fixed probability of containing the true value of the parameter (Neyman, 1937). Some authors argue that the CI defines a set of plausible (or likely) values for parameters. Thus, values within the CIs would be more likely than those outside them. Therefore, we might think of the lower and upper limits as likely lower and upper boundaries for parameters (Cumming, 2014). Consequently, CIs would indicate the precision of the estimate of the parameter, and so the width of a confidence interval would represent the precision of the point estimate of a statistic. Nevertheless, from a frequentist perspective, these interpretations would not be correct. They would be fallacies, given that they would involve “post-data” inferences, that is, reasoning about the parameter from the observed data (Morey et al., 2016). CIs generated from our data do not allow us to make probability statements about parameters. In other words, we cannot know the probability that a particular CI constructed from our data includes the true value. Therefore, it would be incorrect to interpret a CI as the probability that the true value is within the interval (Kline, 2013; Hoekstra et al., 2014; García-Pérez and Alcalá-Quintana, 2016).

On the other hand, CIs might be used to carry out significance testing by determining whether the null value is inside or outside the interval (Kalinowski and Fidler, 2010; Cumming, 2012; García-Pérez and Alcalá-Quintana, 2016). Prior research showed that CIs allow better inference than statistical significance testing in some conditions (Belia et al., 2005; Coulson et al., 2010; Hoekstra et al., 2012). In sum, CIs have been proposed as a possible tool to help reduce NHST misconceptions. However, they are not immune to misinterpretations (Belia et al., 2005; Hoekstra et al., 2014; García-Pérez and Alcalá-Quintana, 2016; Kalinowski et al., 2018).

Finally, meta-analytic studies have gained considerable relevance and prevalence in the most prestigious journals because they synthesize all the evidence available to answer a question based on the results of primary studies (Bauer, 2007). To conduct a meta-analysis, the primary studies have to provide information about ESs and their CIs. A meta-analysis facilitates more precise effect size estimations, it allows researchers to rate the stability of the effects, and it helps them to contextualize the effect size values obtained in their studies. Moreover, the results of a meta-analytic study help to plan future sample sizes by providing the value of the estimated effect size in a specific research context. In addition, meta-analytic studies can provide a definitive answer about the nature of an effect when there are contradictory results (Glass, 1976; Borenstein et al., 2009; Hempel et al., 2013). Nevertheless, meta-analytic studies are not free of bias or misuse (Rothstein et al., 2005; Kicinski, 2013; Ueno et al., 2016). For example, Ferguson and Brannick (2011) analyzed 91 meta-analytic studies published in the American Psychological Association and Association for Psychological Science journals and found that 26 (41%) reported evidence of publication bias. Therefore, researchers and readers of meta-analytic studies (such as practitioner psychologists) should know about methods for detecting these biases (Bauer, 2007; Spring, 2007). In this way, there are checklists to appraise the quality of meta-analytic studies, such as AMSTAR or PRISMA-NMA.

A recent study examined the self-reported statistical practice and knowledge level about effect size statistics and meta-analysis in a sample of Spanish academics psychologists (Badenes-Ribera et al., 2016a). According to the results, the academics mainly said they used NHST for data analysis, and to a lesser degree, effect-size statistics, although the latter were becoming more generalized. In addition, the authors found that the academics had poor knowledge about effect size statistics, graphic displays for meta-analyses (e.g., forest plot and funnel plot), and quality checklists for studies (e.g., PRISMA; CONSORT), which might lead to a misinterpretation of results. Finally, academics with greater knowledge about ES statistics presented a profile closer to good statistical practice and research design.

Our main purpose was to analyze the extension of these results to a different geographical area through a direct or exact replication (Stroebe and Strack, 2014) of the aforementioned study by Badenes-Ribera et al. (2016a). The original research was conducted in Spain, whereas the present study was carried out in Italy. For this purpose, we designed an on-line survey that included the same items as in the original research, and we asked academic psychologists to indicate their level of knowledge about ES, their CIs, and meta-analyses, and how they use them.

In the Italian context, there is no information available about the extension of these topics among academic psychologists. Furthermore, the present work was part of a cross-cultural research project between Spain and Italy about statistical cognition and statistical practice, and it was framed within the line of research on cognition and statistical education that our research group has been developing for many years.

## Methods

### Participants

A non-probabilistic (convenience) sample was used. The sample comprised 159 Italian academic psychologists. Of the 159 Italians participants, 45.9% were men, and 54.1% were women, with a mean age of 47.65 years (SD = 10.47). The mean number of years that the professors had spent in academia was 12.90 (*SD* = 10.21). Regarding Psychology knowledge areas, 18.2% of the academics belonged to the area of Development and Educational Psychology, 17.6% to the area of Clinical and Dynamic Psychology, 17.6% to the area of Social Psychology, 17.6% to the area of General Psychology, 10.7% to the area of Methodology, 10.1% to the area of Neuropsychology, and 8.6% to the area of Work and Organizational Psychology. Regarding the type of university, 86.2% worked in public universities (13.8% private universities). Finally, 84.9% of the participants had been reviewers for scientific journals in the past year.

### Instrument

We used an Italian translation of the questionnaire by Badenes-Ribera et al. (2016b) (see the Appendix in Supplementary Material). Therefore, all the items were translated into Italian by applying the standard back-translation procedure, which involved translations from Spanish to Italian and vice versa (Balluerka et al., 2007).

The first section of the questionnaire included items related to information about sex, age, years of experience as an academic psychologist, Psychology knowledge area, and Type of university (private/public). The second section included a set of items associated with statistical knowledge and statistical practice:

**A. Knowledge and use of statistical terms**, evaluated with four questions:

1. “What terms from the following list are you sufficiently familiar with? (you can choose more than one response): standard deviation, confidence interval, sedimentation graph, forest plot, ANOVA, funnel plot, correlation, meta-analysis, regression analysis, effect size” (see Table 1).
2. “Can you give the name of an effect size statistic?”
3. “If you answered Yes, please specify its name” (open-ended question) (see Table 2).
4. “In your scientific reports, what type of statistics do you use more often?” Likert-type response scale with 5 response ratings that range from 0 = not at all, to 4 = used quite often (see Table 3).

**Table 1:** Statistical terms the participants say they know sufficiently (%) (95% Confidence Interval).

	**Current study (*N* = 159)**	**Spanish academics (*N* = 472) (Badenes-Ribera et al., 2016a)**	**Difference scores between Italian and Spanish academics**
Standard deviation	99.4 (96.5, 99.9)	98.9	0.5 (−2.5, 1.9)
ANOVA	98.7 (95.5, 99.7)	97.5	1.2 (−2.1, 3.4)
Regression analysis	98.1 (94.6, 99.4)	94.5	3.6 (−0.3, 6.4)
Correlation	96.9 (92.9, 98.7)	98.5	−1.6 (−5.7, 0.7)
Confidence Intervals	93.7 (88.8, 96.6)	NA	–
Meta-analysis	92.5 (87.3, 95.6)	86.9	5.6 (−0.3, 10.2)
Effect size	81.8 (75, 87)	87.1	−5.3 (−12.6, 0.9)
Forest plot	17.6 (12.4, 24.2)	11	6.6 (0.6, 13.7)
Funnel plot	13.8 (9.3, 20.1)	7	6.8 (1.6, 13.4)
Sedimentation graphic	8.8 (5.3, 14.2)	45.1	−36.3 (−42, −29.3)

*More than one answer could be selected. %, Percentage. NA, Not asked*.

**Table 2:** Known effect size statistics (%) (95% Confidence Interval).

**Effect size statistics**	**Current Study(*n* = 71)**	**Spanish academics (*n* = 323) (Badenes-Ribera et al., 2016a)**	**Difference scores between Italian and Spanish academics**
Cohen's *d*	66.2 (54.6, 76.1)	70.6	−4.4 (−16.9, 6.8)
η^2^	60.6 (48.9, 71.1)	44	16.6 (3.8, 28.4)
Correlation/Association coefficient (Pearson, Spearman, biserial, Phi, Cramer's *V*)	35.2 (25.1, 46.8)	24.8	10.4 (−0.8, 22.9)
Omega/omega^2^	15.5 (8.9, 25.7)	8.1	7.4 (−0.1, 17.9)
*R*^2^	14.1 (7.8, 24)	9.9	4.2 (−3.1, 14.5)
Hedge's *g*	11.3 (5.8, 20.7)	10.8	0.5 (−6.2, 10.3)
Cohen's *f*/Cohen's *f*^2^	11.3 (5.8, 20.7)	2.8	8.5 (2.5, 18)
Odds Ratio	8.5 (3.9, 17.2)	5.9	2.6 (−2.9, 11.6)
Relative Risk	5.6 (2.2, 13.6)	2.5	3.1 (−1, 11.2)
Cohen's *q*	4.2 (1.5, 11.7)	NR	–
Beta	2.8 (0.8, 9.7)	0.9	1.9 (−0.8, 8.8)
Glass' delta	NR	1.9	–
Number Needed to Treat (NNT)	NR	0.9	–
Wilk's Lambda	NR	0.6	–
Epsilon/Epsilon^2^	NR	0.6	–
Cliff's delta	NR	0.3	–
Common Language (CL)	NR	0.3	–

*The majority of participants reported knowing more than one effect size statistic. %, Percentage. NR, Not Reported*.

**Table 3:** Self-reported Use of statistics (%) (95% Confidence Interval).

	**Used quite often**	**Used a fair amount**	**Used sometimes**	**Used very little**	**Not used**
ANOVA	62.9 (55.2, 70)	20.8 (15.2, 27.7)	10.1 (6.3, 15.7)	3.1 (1.4, 7.2)	3.1 (1.4, 7.2)
Correlation	54.7 (47, 62.3)	25.2 (19.1, 32.4)	14.5 (9.8, 20.8)	1.9 (0.6, 5.4)	3.8 (1.7, 8)
Regression	47.8 (40.2, 55.5)	27 (20.7, 34.4)	17 (11.9, 23.6)	5 (2.6, 9.6)	3.1 (1.4, 7.2)
*T*-tests	27.7 (21.3, 35.1)	37.7 (30.6, 45.5)	22.6 (16.8, 29.8)	7.6 (4.4, 12.7)	4.4 (2.2, 8.8)
Effect size	35.9 (28.8, 43.6)	18.2 (13.6, 25.7)	13.8 (9.3, 20.1)	17 (11.9, 23.6)	15.1 (10.4, 21.5)
Confidence intervals	27.7 (21.3, 35.1)	27.7 (21.3, 35.1)	17.6 (12.5, 24.3)	17 (11.9, 23.6)	10.1 (6.3, 15.7)
Effect size and CIs	25.8 (19.6, 33.1)	21.4 (15.7, 28.4)	17 (11.9, 23.6)	18.9 (13.6, 25.7)	17 (11.9, 23.6)
Exploratory factorial analysis	26.4 (20.2, 33.8)	23.9 (17.9, 31.1)	19.5 (14.1, 26.3)	17 (11.9, 23.6)	13.2 (8.8, 19.4)
Confirmatory factorial analysis	23.3 (17.4, 30.4)	18.9 (13.6, 25.7)	21.4 (15.7, 28.4)	24.5 (18.5, 31.8)	12 (7.8, 17.9)
Structural equations	17.6 (12.5, 24.3)	17 (11.9, 23.6)	16.4 (11.4, 22.9)	22 (16.3, 29.1)	27 (20.7, 34.4)
MANOVA	24.5 (18.5, 31.8)	33.3 (26.5, 41)	21.4 (15.7, 28.4)	14.5 (9.8, 20.8)	6.3 (3.5, 11.2)
Discriminant analysis	3.1 (1.4, 7.2)	11.3 (7.3, 17.2)	22.6 (16.8, 29.8)	37.7 (30.6, 45.5)	24.5 (18.5, 31.8)

*%, Percentage*.

**B. Opinions and self-reported use of meta-analytic studies**, evaluated with two questions (see Table 4):

1. “What type of review do you think has the most credibility and objectivity?” (choose only one response):
  - The narrative review carried out by experts (such as those performed in the “Annual Review”).
  - The quantitative review or meta-analysis.
  - The qualitative review.
2. “Have you read or used a meta-analytic study?”
  - I have never read or used one.
  - Yes: I have read or used 1−2 meta-analytic studies.
  - Yes, I have read or used more than 2 meta-analytic studies.

**Table 4:** Opinion, reading or use of meta-analytic studies (%) (95% Confidence Interval).

	**Current Study (*N* = 159)**	**Spanish academics (*N* = 472) (Badenes-Ribera et al., 2016a)**	**Difference scores between Italian and Spanish academics**
**OPINIONS ABOUT THE REVIEW WITH MOST CREDIBILITY AND OBJECTIVITY**			
The quantitative review or meta-analysis	73.6 (66.2, 79.8)	57.4	16.2 (7.61, 23.9)
The narrative review carried out by experts	16.4 (11.4, 22.9)	34.3	−17.9 (−24.6, −10.24)
The qualitative review	10.1 (6.3, 15.7)	8.3	1.8 (−2.9, 7.9)
**READING OR USE OF META-ANALYTIC STUDIES**			
I have never read or used one	27.7 (21.3, 35.1)	14.4	13.3 (6, 21.2)
I have read or used 1–2 meta-analytic studies	61.6 (53.9, 68.8)	30.1	31.5 (22.7, 39.8)
I have read or used more than 2 meta-analytic studies	10.7 (6.8, 16.5)	55.5	−44.8 (−50.7, −37.5)

*%, Percentage*.

**C. Researcher's behavior**, evaluated with 11 questions related to research design (e.g., estimate a priori sample size, strategies used for it, and so on), reporting on the *p*-value, and interpretation of the *p*-value (see Table 5).

**Table 5:** Researcher profile based on knowing or not knowing the name of effect size statistics (%) (95% Confidence Interval).

**Item**	**Current study**		**Spanish academics (Badenes-Ribera et al**., **2016a****)**		**Difference scores between Italian and Spanish academics on knowing/not knowing the name of effect size statistics**	
	**Not knowing (*n* = 88)**	**Knowing (*n* = 71)**	**Not knowing (*n* = 149)**	**Knowing (*n* = 323)**	**Not knowing**	**Knowing**
**RESEARCHER'S BEHAVIOR**						
Have you read or used a meta-analytic study?						
I have never read or used one	35.2 (26.1, 45.6)	18.3 (11, 28.9)	28.9	7.8	6.3 (−5.6, 18.7)	10.5 (2.5, 21.4)
Yes: I have read or used 1−2 meta-analytic studies	**52.3 (42, 62.4)**	**73.2 (62, 82.2)**	**36.9**	26.9	15.4 (2.3, 27.9)	46.3 (33.9, 56.3)
Yes, I have read or used more than 2 meta-analytic studies	12.5 (7.1, 21)	8.5 (3.9, 17.2)	34.2	**65.3**	−21.7 (−31.3, −10.6)	−56.8 (−63.6, −46.6)
Have you been a reviewer for a scientific journal in the past year?						
No	21.6 (14.3, 31.3)	7 (3.1, 15.5)	**48.3**	29.1	−26.7 (−37.5, −14.2)	−22.1 (−28.6, −12.4)
Yes: 1–2 reviewed articles	25 (17.1, 35)	32.4 (22.7, 43.9)	38.3	33.4	−13.3 (−24.5, −0.8)	−1 (−12.1, 11.5)
Yes: more than 2 reviewed articles	**53.4 (43.1, 63.5)**	**60.6 (48.9, 71.1)**	13.4	**37.5**	40 (27.8, 51)	23.1 (10.3, 34.8)
Have you published an article in a journal indexed in the WoS with JCR impact factor in the past year?						
No	22.7 (15.2, 32.5)	11.3 (5.8, 20.7)	**39.6**	21.7	−16.9 (−27.9, −4.5)	−10.4 (−17.7, −0.1)
Yes: 1–2 published articles	31.8 (23, 42.1)	33.8 (23.9, 45.4)	**39.6**	**43**	−7.8 (−19.7, 5)	−9.2 (−20.6, 3.5)
Yes: more than 2 published articles	**45.5 (35.5, 55.8)**	**54.9 (43.4, 66**)	20.8	35.3	24.7 (12.3, 36.5)	19.6 (6.9, 31.7)
What type of review do you think has the most credibility and objectivity?						
The narrative review carried out by experts	17.1 (10.6, 26.2)	15.5(8.9, 25.6)	**40.9**	31.3	−23.8 (−34.2, −12)	−15.8 (−24.2, −4.5)
The quantitative review or meta-analysis	**65.9 (55.5, 75)**	**83.1 (72.7, 90.1)**	**41.6**	**64.7**	24.3 (11.2, 36.1)	18.4 (6.9, 27.2)
The qualitative review	17.1 (10.6, 26.2)	1.4 (0.3, 7.6)	17.4	4	−0.3 (−9.8, 10.2)	−2.6 (−5.6, 3.8)
Do you know of any checklists for assessing the quality of the research design of a study?						
No	**87.5 (79, 92.9)**	**83.1 (72.7, 90.1)**	**91.9**	**78**	−4.4 (−13.6, 3.2)	5.1 (−6.1, 13.5)
Yes	12.5 (7.1, 21)	16.9 (9.9, 27.3)	8.1	22	4.4 (−3.2, 13.6)	−5.1 (−13.5, 6.1)
In your opinion, what statistical questions or issues related to the study design are currently being debated?						
I don't know	**61.4 (50.9, 70.7)**	26.8 (17.9, 38.1)	**79.9**	**53.9**	−18.5 (−30.4, −6.6)	−27.1 (−37.5, −14.6)
I don't think there are any debates open	9.1 (4.7, 16.9)	4.2 (1.5, 11.7)	2	2.2	7.1 (1.3, 15)	2.1 (−1.5, 9.6)
There is some debate	29.6 (21, 39.8)	**69 (57.5, 78.6)**	18.1	44	11.5 (0.4, 23)	25.1 (12.3, 36)
**RESEARCHER'S METHODOLOGICAL BEHAVIOR**						
When you plan a study, do you estimate a priori the sample size you will need?						
No	15.9 (9.7. 25)	16.9 (9.9, 27.3)	21.5	14.6	−5.6 (−15.1, 5.2)	2.4 (−5.8, 13.3)
Yes	**84.1 (75.1, 90.3)**	**83.1 (72.7, 90.1)**	**78.5**	**85.4**	5.6 (−5.2, 15.1)	−2.4 (−13.3, 5.8)
What kind of strategy do you use when you want to plan the sample size of a study?						
You try to achieve the greatest number of participants possible	25 (17.1, 35)	22.5 (14.4, 33.5)	**33.1**	25.17	−8.1 (−19, 4.3)	−2.5 (−12.1, 9.3)
You use software or tables to estimate the sample size according to the statistical criteria	9.1 (4.7, 16.9)	**32.4 (22.7, 43.9**)	25.2	**34.7**	−16.1 (−22.2, −6.4)	−2.3 (−13.4, 10.3)
You try to make the sample represent the characteristics of the population	**56.8 (46.4, 66.7)**	29.6 (20.2, 41)	**33.8**	**37.41**	23 (10.2, 35.4)	−7.9 (−18.7, 4.6)
You do not use any strategy because it isn't part of your research interests	9.1 (4.7, 16.9)	15.5 (8.9, 25.7)	7.9	2.7	1.2 (−6, 9.6)	12.7 (5.7, 23)
In your opinion, what is the purpose of calculating the statistical power a priori?						
To adjust the significance level or alpha value	28.4 (20, 38.6)	4.2 (1.5, 11.7)	47	33.3	−18.6 (−30.1, −5.7)	−28.9 (−34.9, −20)
To explore the reliability of the scales	10.2 (5.5,18.3)	4.2 (1.5, 11.7)	13.6	4.8	−3.4 (−11.2, 6.1)	−0.7 (−4.8, 7)
To estimate the sample size	**42.1 (32.3, 52.5)**	**76.1 (65, 84.5)**	**39.4**	**61.9**	2.7 (−10.2, 15.3)	14.1 (1.9, 24.2)
Don't know/don't respond	19.3 (12.4, 28.4)	15.5 (8.9, 25.7)	-	-	-	-
In your opinion, obtaining a statistically significant result implies indirectly that the detected effect is important						
No	**89.8 (81.7, 94.5)**	**91.5 (82.8, 96.1)**	45.6	**69.7**	44.2 (32.8, 53.3)	21.9 (11.9, 28.8)
Yes	10.2 (5.5,18.3)	8.5 (3.9, 17.2)	**54.4**	30.3	−44.2 (−53.3, −32.8)	−21.9 (−28.8, −11.9)
When you perform a statistical test, do you consider it a priority to always report the statistical significance obtained?						
No	9.1 (4.7, 16.9)	22.5 (14.4, 33.5)	5.4	3.7	3.7 (−2.9, 12)	18.8 (10.2, 29.9)
Yes, and using expressions such as *p* < 0.05, *p* > 0.05	**68.2 (57.9, 77)**	**40.9 (30.2, 52.5)**	**59.7**	41.8	8.5 (−4.3, 20.4)	1 (−12.9, 11.8)
Yes, and using expressions with the *p*-value of exact probability	22.7 (15.2, 32.5)	**36.6 (26.4, 48.2)**	34.9	**54.5**	−12.2 (−23.1, 0.1)	−17.9 (−29.4, −5)

*%, Percentage; Bold values indicates higher percentage*.

To facilitate cross-references, we administered our questionnaire in the same way and with identical instructions to the original research. That is, the questionnaire was administered online through a CAWI (Computer Assisted Web Interviewing) system.

### Procedure

The e-mail addresses of Italian academic psychologists were found by consulting the websites of Italian universities, resulting in 1,824 potential participants. Of the 1,824 potential participants, 40.5% were men, and 59.5% were women. Regarding Psychology knowledge areas, 23.6% of the academics belonged to the area of Clinical and Dynamic Psychology, 21.9% to the area of General Psychology, 15.1% to the area of Development and Educational Psychology, 14.1% to the area of Social Psychology, 11.7% to the area of Neuropsychology, 6.2% to the area of Methodology, and 7.4% to the area of Work and Organizational Psychology. Finally, regarding the type of university, 88.8% worked in public universities (11.2% private universities).

Potential participants were invited to complete a survey through the use of a CAWI system. A follow-up message was sent 2 weeks later to non-respondents. Individual informed consent was also collected from the academics, along with written consent describing the nature and objective of the study, following the ethical code of the Italian Association for Psychology (AIP) and with adherence to the privacy requirements required by Italian law (Law DL-196/2003). The consent form stated that data confidentiality would be ensured and that participation was voluntary. As regards the ethical standards for research, the study complied with the latest version of the Declaration of Helsinki. The response rate was 8.72%. The data collection was performed from March to May 2015.

### Data analysis

The analysis included descriptive statistics for the variables under evaluation (e.g., frequencies and percentage). We used scoring methods based on the studies by Newcombe (2012) in order to calculate the confidence intervals for percentages. These analyses were performed with the statistical program IBM SPSS v. 20 for Windows.

## Results

Table 1 presents the percentages of participants' responses about their level of knowledge about statistical terms. More than 90% of participants thought they had an adequate knowledge of CIs, analysis of variance, regression analysis, standard deviation, correlation, and meta-analysis. Additionally, more than 80% said they had adequate knowledge about the statistical terms for effect size.

Although more than 90% percent of the participants said they had adequate knowledge about meta-analyses, the statistical terms of forest plot and funnel plot (graphics that usually accompany meta-analytic studies) were rated as being sufficiently known by a very low percentage of the participants, as in the original study. This was especially true of the funnel plot graphics, which are used primarily as a visual aid for detecting publication bias.

Regarding their knowledge about effect size statistics, 82% of the participants stated that they had adequate knowledge about effect sizes. However, only 44.7% (95% CI 37.1, 52.4) (*n* = 71) stated that they knew some effect size statistic. Therefore, as in the original study, there was greater knowledge about the term “effect size” than about the actual use of effect size statistics.

Table 2 shows the effect size statistics known by participants. The most familiar effect size statistics were those that evaluate the differences between the means of the groups analyzed (standardized mean difference), followed by the proportion of variance explained (η^2^) and correlation coefficients.

Table 3 presents the self-reported use of statistics in research reports. Overall, the analysis of variance (ANOVA) was the most widely used statistic in research reports, followed by correlation and regression analysis. In addition, the majority of the participants (52.9%) said they used effect sizes and CIs little (17 % and 18.9%) or not at all (17%) in their statistical reports. Finally, discriminant analysis was utilized the least.

Table 4 presents the opinions and self-reported use of meta-analytic studies. Most of the participants (66.2%) pointed out that meta-analytic studies are the type of review with the most credibility and objectivity, and they said they had used or read a meta-analytic study for their research, as in the original study with Spanish academics.

Table 5 shows the profile of Italian academics according to whether or not they were able to indicate the name of an effect size statistic, compared to the profile of Spanish academics from the original study (Badenes-Ribera et al., 2016a). Overall, in both samples, the academics who gave the name of an effect size statistic had behaviors that were closer to good statistical practices and research design. Therefore, among the academics who gave the name of an effect size statistic, a higher proportion of participants had read or used meta-analytic studies, had been reviewers for scientific journals, had published an article in journals with a JCR impact factor (Journal Citation Reports of WoS), and thought that meta-analytic studies were the type of review with the most credibility. Furthermore, as in the original research, Italian academics who named an effect size statistic performed better methodological practices than the rest of the participants. Thus, fewer of them confused planning the statistical power a priori as a strategy to adjust the significance level or alpha value, and a larger percentage stated that they estimated the a priori sample size, and planned the number of participants using statistical criteria. However, only a third of them followed the statistical recommendations of avoiding *p*-value expressions such as *p* < alpha or *p* > alpha, and used the exact *p*-value instead. Moreover, they said they knew that there was currently open debate about statistical issues or the research design (*n* = 49), unlike in the original research, where most of the participants did not know that there was an open debate on these topics. In fact, most of these participants (73.5% of 49 academics) mentioned some issue associated with criticism of the NHST approach (e.g., interpretation *p*-values, publication bias, false positive, *p*-hacking etc.). For instance, 30.6% cited alternative statistical methods to NHST proposed in the literature (e.g., effect size; Bayesian approach, estimation by confidence intervals), and 14% mentioned the importance of conducting replication studies. By contrast, among academics who did not give the name of an effect size statistic and said they knew about the debate about statistical issues or the research design (*n* = 26), only 42.3% (95% CI 25.5, 61.1) cited an issue associated with criticism of the NHST approach (e.g., interpretation of *p*-values, effect size statistics, Bayesian approach, estimation by confidence interval, and replication). The most prevalent issue mentioned was the debate about quantitative vs. qualitative approaches to conduct research (19.2%).

Finally, in both groups, the majority of the participants said they did not know of any checklist to assess the quality of a study design, a result also found in the original research. In addition, when the participants were asked to mention the name of a checklist, the percentage who actually mentioned the name of at least one checklist was a little lower. Only 12.7% (95% CI 6.8, 22.4) of the participants who named an ES, and 8% (95% CI 3.9, 15.5) of the participants who did not, actually mentioned a checklist. Again, there was a higher self-reported knowledge level than actual knowledge. The checklists mentioned most were the Strobe Statement, PRISMA, CONSORT, Newcastle-Ottawa Scale (NOS), and Journal Articles Reporting Standards (JARS).

In addition, they did not have the practical significance fallacy, that is, the false belief that the *p*-value indicates the importance of the findings, unlike in the original research, where the participants who did not name an effect size statistic had a higher percentage of this misconception about the *p*-value.

## Conclusion and discussion

Our work was a direct replication of the study by Badenes-Ribera et al. (2016a). We found that reporting ES statistics and their CI is becoming generalized, as in the original research. Hence, the majority of participants (54.1%) stated that they used ES statistics a fair amount, and 47.2% of them said that they used CIs for ES statistics a fair amount. Therefore, academic psychologists show some signs of change, after half a century of arguments against NHST and calls to adopt alternative practices. However, changing statistical practice is not easy. For example, contemporary researchers have not been sufficiently trained to adopt methodological alternatives; most of them have been trained in courses focused on calculating and reporting the results of NHST. In addition, the methods needed to calculate ES statistics and their CIs are not available in most statistical software programs.

The effect size statistics most widely known by participants were those from the family of standardized mean differences and η^2^, which are parametric effect size statistics. These findings suggest that the participants did not know, or at least did not mention, the alternatives to parametric effect size statistics, such as non-parametric statistics (e.g., Spearman's correlation), the robust standardized mean difference (trimmed means and Winsorized variances), the number needed to treat, or the probability of superiority (PS) (Erceg-Hurn and Mirosevich, 2008; Keselman et al., 2008; Grissom and Kim, 2012).

Furthermore, standardized mean differences (e.g., Cohen's *d*, Glass' delta, Hedges' *g*,) and effect sizes from the types of correlations (e.g., Pearson's correlation, *R*^2^, η^2^, omega^2^) have been criticized for not showing robustness against outliers or for departing from normality, and instability when statistical assumptions are violated (Algina et al., 2005; Wang and Thompson, 2007; Grissom and Kim, 2012; Kline, 2013; Peng and Chen, 2014). These findings provide more evidence of the need for statistical training programs for academics in order to improve their professional practice and teaching, given that through their teaching activities they will influence many students who will have a professional future in the field of Psychology.

Regarding their opinions about reviews, the majority of the participants considered systematic reviews and meta-analytic studies to be more credible and objective than other types of literature reviews. They also thought they had adequate knowledge about meta-analyses and stated that they used meta-analytic studies in their professional practice. Nevertheless, they acknowledged having poor knowledge about effect sizes and graphic displays for meta-analyses, such as forest plots and funnel plots. Graphic portrayal of results is an important aspect of a meta-analysis and the main tool for presenting results from different studies on the same research topic (Borenstein et al., 2009; Anzures-Cabrera and Higgins, 2010). Thus, forest plots and funnel plots are graphics used in meta-analytic studies to present pooled effect size estimates and publication bias, respectively. In fact, the funnel plot is used as a publication bias detection method in the health sciences (Sterne et al., 2005). In the absence of publication bias, the plot presents a symmetrical inverted funnel; and in the presence of bias, the plot is asymmetrical. However, it should be taken into account that there are other sources that can contribute to the asymmetry of a funnel plot and should not be confused with publication bias, such as heterogeneity, chance, or the choice of the statistics being plotted (Sterne et al., 2011; Badenes-Ribera et al., 2017a). Moreover, the visual inspection of funnel plots to detect publication bias is open to subjective interpretation by researchers (Jin et al., 2015).

In addition, publication bias is common in meta-analytic studies (Ferguson and Brannick, 2011). Therefore, publication bias is an important threat to the validity of meta-analytic studies because meta-analytically derived estimates could be inaccurate, typically overestimated. Consequently, publication bias may distort scientific knowledge about topics related to health and other topics of scientific interest (Rothstein et al., 2005). As Kepes et al. (2014) point out, publication bias has been referred to as “the Achilles' heel of systematic reviews” (Torgerson, 2006), “the kryptonite of evidence-based practice” (Banks and McDaniel, 2011), and the “antagonist of effective policy making” (Banks et al., 2012). Therefore, researchers, academics, and practitioners must have adequate knowledge about the funnel plot, which is a basic tool of meta-analytic studies to detect bias publication and heterogeneity of effect sizes, allowing the reader to appraise whether the results of the meta-analysis reflect an undistorted view of effect sizes (Bauer, 2007). Consequently, training programs in skills to evaluate meta-analyses are needed so that academics can appraise the quality and relevance of available evidence stemming from them (Spring, 2007; Walker and London, 2007).

Regarding the methodological quality checklists, as in the original research, most of the participants said they did not have any knowledge about them. In relation to the above, it should be clarified that there are checklists for primary studies (e.g., CONSORT) and for meta-analytic studies (e.g., AMSTAR or PRISMA-NMA). These checklists are useful for appraising research evidence (Spring, 2007).

Finally, as in the original research, when analyzing the researcher's behavior with regard to methodological practices, the results showed that participants with some knowledge of effect size statistics demonstrated better statistical and research design practices, participated more in the peer review process, and published in high-impact journals. Nevertheless, a large proportion continue to use *p*-values that revolve around the alpha value (as in study by Giofrè et al., 2017), and are not sure why statistical power should be planned a priori. Nevertheless, unlike in the original research, a larger percentage of Italian academics stated they knew there was an open debate about statistical practices and did not commit the significance fallacy. Given what we know about researchers' understanding of significance testing (e.g., misconceptions about the *p*-value), we did not expect Italian academics to be completely aware of this issue. It is possible that the answer to the question about the significance fallacy was largely determined by the undertone that “no” was the right answer, rather than reflecting what they really thought.

In summary, the main similarities observed between the original group and the present group were on the level of (un)knowledge about statistical terms and the use of statistical techniques, given that they seem to know the same statistical terms and techniques, and the statistical techniques they said they do not know and do not use are approximately the same. These findings suggest that both academic groups receive similar statistical and methodological training in their countries. Therefore, to improve the academic training, new statistics programs and textbooks are needed with information about how to calculate and report effect sizes and their confidence intervals, and how to interpret the findings of meta-analytic studies.

On the other hand, the main difference between the two studies was that in the original study, academics with greater knowledge about ES statistics presented a profile closer to good statistical practice and research design, whereas in the present study, the difference between the methodological profile of academics who gave the name of an ES statistic and academics who did not was less clear. In both groups, a profile close to good statistical practice and research design can be observed. This difference between Spanish academics and Italian academics might be explained by the time that passed between one study and the other (nearly 3 years). During that time, the debate about the use, understanding, and abuse of the *p-*value, good statistical practices, and research design was re-opened. In addition, publications on good statistical practices increased (e.g., Cumming, 2014; Nuzzo, 2014; DeCoster et al., 2015; Earp and Trafimow, 2015; Valentine et al., 2015), in addition to editorial journals encouraging researchers to change their statistical practice from reporting NHST to alternatives such as reporting ES and their CIs (e.g., Trafimow and Marks, 2015). The renewed debate, publications, and editorial journals might have influenced the methodological behavior of Italian academics. In fact, a larger percentage of Italian academics than Spanish academics knew about this statistical practice debate.

## Limitations

We acknowledge several limitations of this study. First, the low response rate could have affected the results because, of the 1,824 academic psychologists who received an e-mail with the link to the survey, only 159 participated (8.72%). The low response rate might affect the representativity of the sample and, consequently, the generalizability of the results. In addition, the participants who answered the survey may have felt more confident about their knowledge of statistics than the academics who did not respond. Thus, the findings might overestimate the knowledge level and self-reported use of ES, CIs, and meta-analysis, and the extent of the statistical reform in Italian academic psychologists. Furthermore, some participants do not use quantitative methods at all, making them more reluctant to respond.

Another limitation is that we asked respondents to use Likert-scale items to capture whether they felt adequately knowledgeable about a specific statistic. However, this question and response format do not allow us to know whether the respondents are deceiving themselves. That is, these questions are actually measuring their belief and confidence about their knowledge, but their actual knowledge might be quite different. Several studies on NHST indicate that a large number of academics in various degrees have incorrect knowledge about statistical significance or CIs (e.g., Hoekstra et al., 2014; Badenes-Ribera et al., 2015, 2016a). This bias could be controlled in future research by formulating the questions (e.g., about the correct interpretation of a specific forest plot, funnel plot, effect size, or regression analysis) using a format with three or four responses or asking an open-ended question. These response formats make it possible to assess knowledge about statistical terms, which would provide more information.

Another limitation related to the items on the questionnaire used is that, because the questionnaire targets multiple issues, the information about each of these topics is rather limited. That is, some items on the questionnaire are quite generic. However, because the purpose of this work was to replicate the study by Badenes-Ribera et al. (2016a), we maintained the question format to facilitate the comparison of the two studies. Examples of items with problems are: the questions about CIs only collect information about whether the participants think they understand what CIs are and whether they are used, but they do not detect whether the participants correctly interpret the CIs. The question “Have you read or used a meta-analytic study?” would be much more informative as: “How many meta-analyses do you think you've read in your career?” or something along these lines. The question “What kind of strategy do you use when you want to plan the sample size of a study?” should have been an open question, which would have been far more informative. Finally, the question “In your opinion, what statistical questions or issues related to the study design are currently being debated?” could have been more specific, directly asking whether the respondents know about the debate on the criticisms of the NHST approach, or any alternative statistical methods to NHST. In this case, we thought social desirability might affect the participants' answers, and we believed it was better to ask first about the existence of an open debate, and then about what issues are being debated.

Indeed, it is possible that there was a social desirability effect, which can occur when data are collected through self-report questionnaires. For instance, as in the original study, the percentage of participants who said they had adequate knowledge about the effect size term was higher than the percentage of participants who could name an ES statistic, and the percentage of participants who said they could name an ES statistic was higher than the percentage of participants who actually did so. The same pattern of answers was noted when we asked participants if they knew of a checklist for assessing the research design of a study and then they were asked to mention the name of one. The percentage of response was a little lower on the latter question.

Nevertheless, all our findings agree with the study carried out with Spanish academic psychologists (Badenes-Ribera et al., 2016b), practitioner psychologists (Badenes-Ribera et al., 2017b), and prior research analyzing the use of effect size statistics and their confidence intervals in scientific journals with impact (Fritz et al., 2012; Peng et al., 2013; Tressoldi et al., 2013; Giofrè et al., 2017). In these studies, a majority use of the NHST procedure can be noted, as well as an increase in the use of effect size statistics (such as Cohen's *d* and the *R*^2^/η^2^) and CIs for ES.

All of these findings indicate that, although the use of effect-size estimators is becoming generalized, several inadequate statistical practices persist, for instance, using statistical inference techniques (e.g., ANOVA and *T*-test) without reporting effect size estimations and/or confidence intervals for the effect size estimated, and the use of relative (or inexact) *p-*values.

Changing statistical practice is not easy. This change requires the deinstitutionalization of NHST, which in turn, needs cultural-cognitive, normative, and regulative preconditions (Orlitzky, 2012). To do so, changes must be made in undergraduate and graduate training (Giofrè et al., 2017), which focus on calculating and reporting the NHST. These courses should include alternatives to traditional statistical approaches. In addition, the most widely used statistical software programs (e.g., SPSS) do not include the calculation of CIs for ES among their options, or the calculation of robust effect sizes, and so they should be updated to facilitate change in statistical practice. There are several free and open statistical software programs (e.g., Jamovi) to assist researchers in estimating ES and constructing confidence intervals for ES estimates computed from sample data (this software is available from https://www.jamovi.org). Moreover, there are several websites that allow the computation of ES estimators and their CIs (for more information see Fritz et al., 2012; Peng et al., 2013).

In addition, the change requires journal editors and reviewers to clearly and decisively ask authors to report effect size statistics and their confidence intervals. Journal editors and reviewers are crucial in checking the practices proposed are adopted by Peng et al. (2013) and Giofrè et al. (2017).

In short, good statistical practice, good research design and correct interpretation of the results in a context are essential components of a good scientific practice for the accumulation of a valid scientific knowledge (Wasserstein and Lazar, 2016). There remains a clear need to raise awareness among professionals and academics in Psychology about these guidelines, but especially to promote the education and training in the use of these practices in order to build better science. Evidence-based practices require professionals to critically assess the findings from psychological research. In order to do so, training in statistical concepts, research design methodology, and results of statistical inference tests has to be provided.

## Author contributions

LB-R, DF-N, NI, AB-C, and CL conceptualized and wrote the paper.

### Conflict of interest statement

The authors declare that the research was conducted in the absence of any commercial or financial relationships that could be construed as a potential conflict of interest.

## References

[B1] Agnoli F. Wicherts J. M. Veldkamp C. L. S. Albiero P. Cubelli R. (2017). Questionable research practices among Italian research psychologists. PLoS ONE 12:e0172792. 10.1371/journal.pone.0172792 28296929 PMC5351839

[B2] Algina J. Keselman H. J. Penfield R. D. (2005). An alternative to Cohen's standardized mean difference effect size: a robust parameter and confidence interval in the two independent groups case. Psychol. Methods 10, 17–328. 10.1037/1082-989X.10.3.317 16221031

[B3] Allison D. B. Brown A. W. George B. J. Kaiser K. A. (2016). Reproducibility: a tragedy of errors. Nature 530, 27–29. 10.1038/530027a 26842041 PMC4831566

[B4] American Psychological Association (2010). Publication Manual of the American Psychological Association, 6th Edn Washington, DC: American Psychological Association.

[B5] American Educational Research Association (2006). Standards for reporting on empirical social science research in AERA publications. Educ. Res. 35, 33–40. 10.3102/0013189X035006033

[B6] Anzures-Cabrera J. Higgins J. P. T. (2010). Graphical displays for meta-analysis: an overview with suggestions for practice. Res. Synth. Methods 1, 66–80. 10.1002/jrsm.6 26056093

[B7] Appelbaum M. Cooper H. Kline R. B. Mayo-Wilson E. Nezu A. M. Rao S. M. (2018). Journal article reporting standards for quantitative research in psychology: the APA Publications and Communications Board task force report. Am. Psychol. 73, 3–25. 10.1037/amp0000191 29345484

[B8] Badenes-Ribera L. Frias-Navarro D. Bonilla-Campos A. (2017a). Biaix de publicació en meta-anàlisi: revisió dels mètodes de detecció i avaluació [Publication bias in meta-analysis: review and evaluation of methods of detection]. Anu. Psicol. 18, 13–30. 10.7203/anuari.psicologia.18.1.13

[B9] Badenes-Ribera L. Frias-Navarro D. Bonilla-Campos A. (2017b). Un estudio exploratorio sobre el nivel de conocimiento sobre el tamaño del efecto y meta-análisis en psicólogos profesionales españoles [An exploratory study on the level of knowledge about effect size and meta-analysis in Spanish practtioner psychologists]. Eur. J. Invest. Health Psychol. Educ. 7, 111–122. 10.30552/ejihpe.v7i2.200

[B10] Badenes-Ribera L. Frias-Navarro D. Iotti B. Bonilla-Campos A. Longobardi C. (2016a). Misconceptions of the *p*-value among Chilean and Italian academic psychologists. Front. Psychol. 7:1247. 10.3389/fpsyg.2016.01247 27602007 PMC4993781

[B11] Badenes-Ribera L. Frias-Navarro D. Monterde-i-Bort H. Pascual-Soler M. (2015). Interpretation of the *p*-value. A national survey study in academic psychologists from Spain. Psicothema 27, 290–295. 10.7334/psicothema2014.283 26260938

[B12] Badenes-Ribera L. Frias-Navarro D. Pascual-Soler M. Monterde-i-Bort H. (2016b). Level of knowledge of the effect size statistics, confidence interval and meta-analysis in Spanish academic psychologists. Psicothema 26, 448–456. 10.7334/psicothema2016.24 27776615

[B13] Balluerka N. Gorostiaga A. Alonso-Arbiol I. Haranburu M. (2007). La adaptación de instrumentos de medida de unas culturas a otras: una perspectiva práctica. [Adapting measuring instruments across cultures: a practical perspective]. Psicothema 19, 124–133. 17295994

[B14] Banks G. C. Kepes S. Banks K. P. (2012). Publication bias: the antagonist of meta-analytic reviews and effective policymaking. Educ. Eval. Policy Anal. 34, 259–277. 10.3102/0162373712446144

[B15] Banks G. C. McDaniel M. A. (2011). The kryptonite of evidence based I-O psychology. Ind. Organ. Psychol/ Perspect. Sci. Pract. 4, 40–44. 10.1111/j.1754-9434.2010.01292.x

[B16] Bauer R. M. (2007). Evidence-based practice in psychology: implications for research and research training. J. Clin. Psychol. 63, 685–694. 10.1002/jclp.20374 17551939

[B17] Belia S. Fidler F. Williams J. Cumming G. (2005). Researchers misunderstand confidence intervals and standard error bars. Psychol. Methods 10, 389–396. 10.1037/1082-989X.10.4.389 16392994

[B18] Borenstein M. Hedges L. V. Higgins J. P. T. Rothstein H. (2009). Introduction to Meta-Analysis. Chichester, UK: Wiley 10.1002/9780470743386

[B19] Cohen J. (1988). Statistical Power Analysis for the Behavioral Sciences, 2nd Edn Jersey City, NJ: Lawrence Erlbaum.

[B20] Coulson M. Healey M. Fidler F. Cumming G. (2010). Confidence intervals permit, but do not guarantee, better inference than statistical significance testing. Front. Psychol. 1:26 10.3389/fpsyg.2010.00026 21607077 PMC3095378

[B21] Cumming G. (2012). Understanding the New Statistics: Effect Sizes, Confidence Intervals, and Meta-Analysis. New York, NY: Routledge.

[B22] Cumming G. (2014). The new statistics: why and how. Psychol. Sci. 25, 7–29. 10.1177/0956797613504966 24220629

[B23] DeCoster J. Sparks E. A. Sparks J. C. Sparks G. G. Sparks C. W. (2015). Opportunistic biases: their origins, effects, and an integrated solution. Am. Psychol. 70, 499–514. 10.1037/a0039191 26348333

[B24] Earp B. D. Trafimow D. (2015). Replication, falsification, and the crisis of confidence in social psychology. Front. Psychol. 6:621. 10.3389/fpsyg.2015.00621 26042061 PMC4436798

[B25] Eich E. (2014). Business not as usual. Psychol. Sci. 25, 3–6. 10.1177/0956797613512465 24285431

[B26] Erceg-Hurn D. M. Mirosevich V. M. (2008). Modern robust statistical methods: an easy way to maximize the accuracy and power of your research. Am. Psychol. 63, 591–601. 10.1037/0003-066X.63.7.591 18855490

[B27] Fanelli D. (2009). How many scientists fabricate and falsify research? A systematic review and meta-analysis of survey data. PLoS ONE 4:e5738. 10.1371/journal.pone.0005738 19478950 PMC2685008

[B28] Ferguson C. J. Brannick M. T. (2011). Publication bias in psychological science: prevalence, methods for identifying and controlling, and implications for the use of meta-analyses. Psychol. Methods 17, 120–128. 10.1037/a0024445 21787082

[B29] Fritz C. O. Morris P. E. Richler J. J. (2012). Effect size estimates: current use, calculations, and interpretation. J. Exp. Psychol. Gen. 141, 2–18. 10.1037/a0024338 21823805

[B30] Gadbury G. L. Allison D. B. (2014). Inappropriate fiddling with statistical analyses to obtain a desirable *p*-value: tests to detect its presence in published literature. PLoS ONE 7:e46363. 10.1371/journal.pone.0046363 23056287 PMC3466248

[B31] García-Pérez M. Alcalá-Quintana R. (2016). The interpretation of Scholars' interpretations of confidence intervals: criticism, replication, and extension of Hoekstra et al. (2014). Front. Psychol. 7:1042. 10.3389/fpsyg.2016.01042 27458424 PMC4937803

[B32] Giofrè D. Cumming G. Fresc L. Boedker I. Tressoldi P. (2017). The influence of journal submission guidelines on authors' reporting of statistics and use of open research practices. PLoS ONE 12:e0175583. 10.1371/journal.pone.0175583 28414751 PMC5393581

[B33] Glass G. V. (1976). Primary, secondary, and meta-analysis of research. Educ. Res. 5, 3–8. 10.3102/0013189X005010003

[B34] Grissom R. J. Kim J. J. (2012). Effect Sizes for Research. New York, NY: Routledge.

[B35] Hempel S. Miles J. N. Booth M. J. Wang Z. Morton S. C.. (2013). Risk of bias: a simulation study of power to detect study-level moderator effects in meta-analysis. Syst. Rev. 28:107. 10.1186/2046-4053-2-107 24286208 PMC4219184

[B36] Hoekstra R. Johnson A. Kiers H. A. L. (2012). Confidence intervals make a difference: effects of showing confidence intervals on inferential reasoning. Educ. Psychol. Meas. 72, 1039–1052. 10.1177/0013164412450297

[B37] Hoekstra R. Morey R. D. Rouder J. N. Wagenmakers E. (2014). Robust misinterpretation of confidence intervals. Psychon. Bull. Rev. 21, 1157–1164. 10.3758/s13423-013-0572-3 24420726

[B38] Jin Z.-C. Zhou X.-H. He J. (2015). Statistical methods for dealing with publication bias in meta-analysis. Stat. Med. 34, 343–360. 10.1002/sim.6342 25363575

[B39] Kalinowski P. Fidler F. (2010). Interpreting significance: the differences between statistical significance, effect size, and practical importance. Newborn Infant Nurs. Rev. 10, 50–54. 10.1053/j.nainr.2009.12.007

[B40] Kalinowski P. Lai J. Cumming G. (2018). A cross-sectional analysis of students' intuitions when interpreting CIs. Front. Psychol. 9:112. 10.3389/fpsyg.2018.00112 29527180 PMC5829532

[B41] Kepes S. Banks G. C. Oh I. S. (2014). Avoiding bias in publication bias research: the value of “null” findings. J. Bus. Psychol. 29, 183–203. 10.1007/s10869-012-9279-0

[B42] Keselman H. J. Algina J. Lix L. M. Wilcox R. R. Deerin K. N. (2008). A generally robust approach for testing hypotheses and setting confidence intervals for effect sizes. Psychol. Methods 13, 110–129. 10.1037/1082-989X.13.2.110 18557681

[B43] Kicinski M. (2013). Publication bias in recent meta-analyses. PLoS ONE 8:e81823. 10.1371/annotation/51ecf224-b045-4424-8beb-de155769d429 24363797 PMC3868709

[B44] Kline R. B. (2013). Beyond Significance Testing: Statistic Reform in the Behavioral Sciences. Washington, DC: American Psychological Association.

[B45] Krueger J. I. Heck P. R. (2017). The heuristic value of p in inductive statistical inference. Front. Psychol. 8:908. 10.3389/fpsyg.2017.00908 28649206 PMC5465841

[B46] Lakens D. (2013). Calculating and reporting effect sizes to facilitate cumulative science: a practical primer for *t*-tests and ANOVAs. Front. Psychol. 4:863. 10.3389/fpsyg.2013.00863 24324449 PMC3840331

[B47] Maxwell S. E. Lau M. Y. Howard G. S. (2015). Is psychology suffering from a replication crisis? What does “failure to replicate” really mean? Am. Psychol. 70, 487–498. 10.1037/a0039400 26348332

[B48] Morey R. D. Hoekstra R. Rouder J. N. Lee M. D. Wagenmakers E.-J. (2016). The fallacy of placing confidence in confidence intervals. Psychon. Bull. Rev. 23, 103–123. 10.3758/s13423-015-0947-8 26450628 PMC4742505

[B49] Newcombe R. G. (2012). Confidence Intervals for Proportions and Related Mesuares of Effect Size. Boca Raton, FL: CRC Press.

[B50] Neyman J. (1937). Outline of a theory of statistical estimation based on the classical theory of probability. Philos. Trans. R. Soc. Lond. A Math. Phys. Sci. 236, 333–380. 10.1098/rsta.1937.0005

[B51] Nuzzo R. (2014). Statistical errors: *P*-values, the “gold standard” of statistical validity, are not as reliable as many scientists assume. Nature 130, 150–152. 10.1038/506150a

[B52] Open Science Collaboration (2015). Estimating the reproducibility of psychological science. Science 349:aac4716 10.1126/science.aac4716 26315443

[B53] Orlitzky M. (2012). How can significance tests be deinstitutionalized? Organ. Res. Methods 15, 199–228. 10.1177/1094428111428356

[B54] Peng C. Y. J. Chen L. T. (2014). Beyond Cohen's d: alternative effect size measures for between subject designs. J. Exp. Educ. 82, 22–50. 10.1080/00220973.2012.745471

[B55] Peng C. Y. J. Chen L. T. Chiang H. Chiang Y. (2013). The impact of APA and AERA guidelines on effect size reporting. Educ. Psychol. Rev. 25, 157–209. 10.1007/s10648-013-9218-2

[B56] Perezgonzalez J. D. (2015). The meaning of significance in data testing. Front. Psychol. 6:1293. 10.3389/fpsyg.2015.01293 26379607 PMC4550752

[B57] Rosnow R. L. Rosenthal R. (2009). Effect sizes: why, when, and how to use them. J. Psychol. 217, 6–14. 10.1027/0044-3409.217.1.6

[B58] Rothstein H. R. Sutton A. J. Borenstein M. (2005). Publication Bias in Meta-analysis: Prevention, Assessment and Adjustments. Chichester, UK: Wiley.

[B59] Spring B. (2007). Evidence-based practice in clinical psychology: what it is, why it matters; what you need to know. J. Clin. Psychol. 63, 611–631. 10.1002/jclp.20373 17551934

[B60] Sterne J. A. Gavaghan D. Egger M. (2005). The funnel plot, in Publication Bias in Metaanalysis: Prevention, Assessment and Adjustments, eds Rothstein H. R. Sutton A. J. Borenstein M. (Chichester, UK: Wiley), 75–98.

[B61] Sterne J. A. Sutton A. J. Ioannidis J. P. A. Terrin N. Jones D. R. Lau J. (2011). Recommendations for examining and interpreting funnel plot asymmetry in meta-analyses of randomised controlled trials. BMJ 342:d4002 10.1136/bmj.d4002 21784880

[B62] Stroebe W. Strack F. (2014). The alleged crisis and the illusion of exact replication. Perspect. Psychol. Sci. 9, 59–71. 10.1177/1745691613514450 26173241

[B63] Sun S. Pan W. Wang L. L. (2010). A comprehensive review of effect size reporting and interpreting practices in academic journals in education and psychology. J. Educ. Psychol. 10, 989–1004. 10.1037/a0019507

[B64] Torgerson C. J. (2006). Publication bias: the Achilles' heel of systematic reviews? Brit. J. Educ. Stud. 54, 89–102. 10.1111/j.1467-8527.2006.00332.x

[B65] Trafimow D. Marks M. (2015). Editorial. Basic Appl. Soc. Psychol. 37, 1–2. 10.1080/01973533.2015.1012991

[B66] Tressoldi P. E. Giofré D. Sella F. Cumming G. (2013). High impact = high statistical standards? Not necessarily so. PLoS ONE 8:e56180 10.1371/journal.pone.0056180 23418533 PMC3571951

[B67] Ueno T. Fastrich G. M. Murayama K. (2016). Meta-analysis to integrate effect sizes within an article: possible misuse and Type I error inflation. J. Exp. Psychol. Gen. 145, 643–654. 10.1037/xge0000159 27019021

[B68] Valentine J. C. Aloe A. M. Lau T. S. (2015). Life after NHST: how to describe your data without “p-ing” everywhere. Basic Appl. Soc. Psychol. 37, 260–273. 10.1080/01973533.2015.1060240

[B69] Walker B. B. London S. (2007). Novel tools and resources for evidence-based practice in Psychology. J. Clin. Psychol. 63, 633–642. 10.1002/jclp.20377 17551935

[B70] Wang Z. Thompson B. (2007). Is the Pearson R^2^ biased, and if so, what is the best correction formula? J. Exp. Educ. 75, 109–125. 10.3200/JEXE.75.2.109-125

[B71] Wasserstein R. L. Lazar N. A. (2016). The ASA's statement on *p*-values: context, process, and purpose. Am. Stat. 70, 129–133. 10.1080/00031305.2016.1154108

[B72] Wicherts J. M. Veldkamp C. L. S. Augusteijn H. E. M. Bakker M. van Aert R. C. M. van Assen M. A. L. M. (2016). Degrees of freedom in planning, running, analyzing, and reporting psychological studies: a checklist to avoid p-hacking. Front. Psychol. 7:1832. 10.3389/fpsyg.2016.01832 27933012 PMC5122713

