# Supplementary material for: Perceived Statistical Knowledge Level and Self-Reported Statistical Practice Among Academic Psychologists
Source: Front Psychol. 2018 Jun 22;9:996. doi: 10.3389/fpsyg.2018.00996 (PMC6024681; doi:10.3389/fpsyg.2018.00996)
Supplement: Supplementary file 1 [file Table_1.DOCX]

# *1. Age:

# *2. Sex:

 Men

 Women

# *3. Please, indicate the name of your knowledge area:

*4. How many years have you been working as a university professor (researcher)?

Write the number

# *5. Your institution is:

 Public

 Private

# *6. Have you published an article in a journal indexed in the WoS with JCR impact factor in the past year?:

##  No

 Yes: 1-2 published articles

 Yes: more than 2 published articles

# *7. Have you been a reviewer for a scientific journal in the past year?

##  No

 Yes: 1-2 reviewed articles

 Yes: more than 2 reviewed articles

# *8. What type of review do you think has the most credibility and objectivity? (CHOOSE ONLY ONE RESPONSE):

 The narrative review carried out by experts (such as those performed in the "Annual Review")

 The quantitative review or meta-analysis

 The qualitative review

# *9. ¿ Do you know of any checklist for assessing the quality of the research design of a study?

##  No

 Yes, Please indicate its name:

# *10. Please indicate what terms from the following list you are sufficiently familiar with (you can choose more than one response):

Standard deviation



Confidence Intervals





Sedimentation graph







Forest Plot

ANOVA

Funnel Plot



Correlation









Meta­analysis

Regression analysis



Effect size

# *11. In your opinion, what statistical questions or issues related to the study design are currently being debated?

 I don’t know

 I don’t think there are any debates open

 There is some debate. Please, indicate what questions or issues are debated:



# *12. Have you read or used a meta-analytic study?

 I have never read or used one

 Yes: I have read or used 1 -2 meta-analytic studies

 Yes: I have read or used more than 2 meta-analytic studies

# *13. When you plan a study, do you estimate a priori the sample size you will need?

##  No

 Yes

# *14. What kind of strategy do you use when you want to plan the sample size of a study?

 You try to achieve the greatest number of participants possible

 You use software or tables to estimate the sample size according to the statistical criteria

 You try to make the sample represent the characteristics of the population

 You do not use any strategy because it isn’t part of your research interests

# *15. In your opinion, what is the purpose of calculating the statistical power a priori?

 To adjust the significance level or alpha value

 To explore the reliability of the scales

 To estimate the sample size

 Don’t know/don’t respond:

# *16. In your opinion, obtaining a statistically significant result indirectly implies that the detected effect is important:

##  No

 Yes

# *17. When you perform a statistical test, do you consider it a priority to always report the statistical significance obtained?:

##  No

 Yes, and using expressions such as *p* < 0.05, *p* > 0.05

 Yes, and using expressions with the *p* value of exact probability

# *18. In your research reports, what types of statistics do you use more often? :

|  | Used quite often | Used a fair amount | Used sometimes | Used very little | Not used |
| --- | --- | --- | --- | --- | --- |
| Student’s t test |  |  |  |  |  |
| Analysis of Variance (ANOVA) |  |  |  |  |  |
| Regression Analysis |  |  |  |  |  |
| Correlation |  |  |  |  |  |
| Discriminant Analysis |  |  |  |  |  |
| Exploratory Factorial Analysis |  |  |  |  |  |
| Confirmatory Factorial Analysis |  |  |  |  |  |
| Structural Equation Models |  |  |  |  |  |
| Effect size |  |  |  |  |  |
| Confidence Intervals |  |  |  |  |  |
| Multivariate models (MANOVA) |  |  |  |  |  |
| Effect size and Confidence Intervals |  |  |  |  |  |

# *19. Can you give the name of an ES statistic?:

 I do not know

 Yes. Please, specify its name: (open-ended question):

Thank you for completing our survey.

This research is part of a study evaluating the use of statistics.

Your responses will be confidential. No individual responses will be identified.

Thank you very much
